# Genetic and economic benefits of selection based on performance recording and genotyping in lower tiers of multi-tiered sheep breeding schemes

**DOI:** 10.1186/s12711-016-0281-2

**Published:** 2017-01-17

**Authors:** Bruno F. S. Santos, Julius H. J. van der Werf, John P. Gibson, Timothy J. Byrne, Peter R. Amer

**Affiliations:** 1 AbacusBio Limited, PO Box 5585, Dunedin, 9058 New Zealand; 2 School of Environmental and Rural Science, University of New England, Armidale, NSW Australia; 3 Cooperative Research Centre for Sheep Industry Innovation, Armidale, NSW Australia

## Abstract

**Background:**

Performance recording and genotyping in the multiplier tier of multi-tiered sheep breeding schemes could potentially reduce the difference in the average genetic merit between nucleus and commercial flocks, and create additional economic benefits for the breeding structure.

**Methods:**

The genetic change in a multiple-trait breeding objective was predicted for various selection strategies that included performance recording, parentage testing and genomic selection. A deterministic simulation model was used to predict selection differentials and the flow of genetic superiority through the different tiers. Cumulative discounted economic benefits were calculated based on trait gains achieved in each of the tiers and considering the extra revenue and associated costs of applying recording, genotyping and selection practices in the multiplier tier of the breeding scheme.

**Results:**

Performance recording combined with genomic or parentage information in the multiplier tier reduced the genetic lag between the nucleus and commercial flock by 2 to 3 years. The overall economic benefits of improved performance in the commercial tier offset the costs of recording the multiplier. However, it took more than 18 years before the cumulative net present value of benefits offset the costs at current test prices. Strategies in which recorded multiplier ewes were selected as replacements for the nucleus flock did modestly increase profitability when compared to a closed nucleus structure. Applying genomic selection is the most beneficial strategy if testing costs can be reduced or by genotyping only a proportion of the selection candidates. When the cost of genotyping was reduced, scenarios that combine performance recording with genomic selection were more profitable and reached breakeven point about 10 years earlier.

**Conclusions:**

Economic benefits can be generated in multiplier flocks by implementing performance recording in conjunction with either DNA pedigree recording or genomic technology. These recording practices reduce the long genetic lag between the nucleus and commercial flocks in multi-tiered breeding programs. Under current genotyping costs, the time to breakeven was found to be generally very long, although this varied between strategies. Strategies using either genomic selection or DNA pedigree verification were found to be economically viable provided the price paid for the tests is lower than current prices, in the long-term.

## Background

In most commercial sheep production systems, improvement of genetic merit is limited to outside sire purchases. In general, selection of ewes in commercial flocks is driven by conformation traits such as soundness and constitution, and sometimes, based on the size of the litter in which the replacement candidate was born. While there is limited scope for selection among ewes within commercial sheep flocks due to limited opportunities to undertake voluntary culling, there may be value in selecting future commercial sires and dams in the multiplier tier of multi-tiered breeding structures composed of nucleus, multiplier and commercial tiers. Performance recording of candidates in the multiplier tier could potentially reduce the difference in the average genetic merit between nucleus and commercial tiers, normally referred to as genetic lag, creating additional economic benefits for the breeding structure. In a typical multiplier flock, selection based on performance brings complexity and can involve substantial costs [1]. However, in commercially integrated multi-tiered breeding structures, sufficient value may be captured to offset these costs, particularly if performance recording in large-scale flocks is facilitated by technology such as electronic identification tools (EID), which ensure reliable individual identification and allow automation for accurate performance data recording [2].

There is also opportunity to exploit recent advances in molecular genetics technology. For instance, DNA parentage testing allows the combination of information from an individual’s relatives and its own phenotypic records to provide the basis for prediction of genetic merit [3]. Meuwissen et al. [4] proposed genomic selection (GS), which enables selection decisions to be made early in the life of animals [5], with highest benefits for traits that are more difficult to measure and have low heritability, or are recorded late in life [6]. For GS to be accurate, it is necessary to record large amounts of phenotypic data on genotyped animals [7]. Traits recorded in the multiplier tier could contribute to the reference population needed for genomic prediction, increasing the overall accuracy of selection within the breeding scheme.

Previous studies have estimated the potential benefits of DNA testing in nucleus sheep breeding [6, 8–10]. However, these studies did not model the impact of implementation of performance recording combined with DNA testing in the multiplier tier of multi-tiered breeding schemes. Lack of estimates of the economic benefit of such technologies prevents the identification of a price point for DNA testing at which implementation becomes profitable in multi-tiered breeding schemes.

In the absence of selection in lower tiers, the genetic lag between the nucleus and multiplier, and also between the multiplier and commercial tiers is typically two generations of genetic progress [11–13]. This assumes that all rams used in the lower tiers are obtained from random progeny selected in the tier immediately above, and that no ewe transfer occurs between the tiers. Supplying improved breeding males to the commercial flock by recording the multiplier tier could consequently reduce the genetic lag relative to the nucleus. The hypothesis of this study is that benefits arise from the selection differential created in the multiplier tier through selection based on breeding values and/or genomic prediction when selecting rams for transfer to the commercial tier, and also when selecting replacement ewes from the multiplier to the nucleus tier.

The objective of this study was to quantify production and economic benefits obtained in the commercial tier of a multi-tiered breeding scheme after the introduction of performance recording and DNA parentage testing or genomic selection in the multiplier tier. A deterministic model was developed to evaluate different selection decisions based on combinations of trait recording, genotyping strategies and ewe replacement policies.

## Methods

### Overview

In this study, we calculated the additional economic benefits from improved performance of commercial animals due to recording and DNA testing in the multiplier tier, relative to a breeding structure without performance recording in the multiplier tier. We developed a simulation model for an integrated three-tier structure to estimate the overall benefits of genetic gain over time. Selection differentials were calculated for traits within a multiple trait breeding objective using selection index theory. The dissemination of this genetic superiority throughout the population was predicted based on gene flow methodology, as developed by Bichard [11]. We developed a simulation model which predicts trait-specific estimates of genetic merit in each cohort (*tier* × *sex* × *age group*) and calculates discounted genetic expression (DGE) coefficients used to quantify the timing and frequency of expressions of the genetic superiority that flows through to the commercial tier. Trait heritabilities, phenotypic and genetic variances and correlations, along with descriptions of the numbers of records on individual selection candidates and their relatives were used in the selection index model developed by van der Werf [14]. The total extra revenue and associated costs of applying recording and selection practices in the multiplier tier of the breeding scheme were calculated based on trait DGE coefficients and economic values. The marginal benefits of performance recording and genotyping for various scenarios were then compared with a base scenario, where there was no performance recording, parentage assignment, or genomic selection in the multiplier tier of the breeding scheme.

### Structure of the breeding scheme

The study was based on the structure of the sheep industry in New Zealand. The breeding scheme underlying most of these systems is a multi-tiered structure, which normally involves the nucleus (ram breeder) and the commercial tier, and in some situations, a multiplier tier. Table 1 presents key parameters related to the modelled breeding scheme that supports a large commercial tier of 180,000 ewes.

**Table 1:** Parameters describing the structure and performance of different tiers within the three-tiered breeding scheme

Parameter	Unit	Tier within the breeding program		
		Nucleus	Multiplier	Commercial
Flock breeding ewes	Head	826	7000	180,000
Flock breeding rams	Head	10	70	1800
Ewes mated to terminal sires	%	0	0	20
Ewes/ram	Head	80	100	100
Ewe replacement rate	%	35	35	30
Mixed age ewe lambing rate^a^	%	210	190	165
Ewe lamb lambing rate	%	100	90	80
Lamb survival	%	79	79	82
Weaning rate	%	166	150	135
Lambs sold as stores^b^	%	–	–	20

^a^Lambing percentage of ewes of 2 years old or older, per ewe mated

^b^Slaughter lambs sold to be finished off farm

### Recursive prediction of genetic merit in the nucleus and multiplier tiers

Recursive equations were used to calculate the average genetic merit in the nucleus and multiplier tiers. The resulting equations were functions of genetic merit of animals born in previous years and corresponding selection differentials. The average genetic merit of offspring born in year *y* for animals in tier *T* of the breeding structure, for trait *j*, was calculated as:

$$O_{y,T}^{j} = \frac{{S_{y,T}^{j} + D_{y,T}^{j} }}{2},$$

where $S_{y,T}^{j}$ and $D_{y,T}^{j}$ are the average genetic merit of the sires and dams of the offspring, respectively. These in turn were calculated as:

$$\begin{aligned} S_{y,T}^{j} & = \phi_{y,T = N} \cdot \left[ {\sum\limits_{t = 1}^{6} {\rho_{t, T = N, s = m} \cdot \left( {O_{y - t, T = N}^{j} +\Delta _{y - t, T = N,s = m}^{j} } \right)} } \right] \\ & \quad + \left( {1 - \phi_{y,T = N} } \right) \cdot \left[ {\sum\limits_{t = 1}^{6} {\rho_{t, T = M, s = m} \cdot \left( {O_{y - t, T = M}^{j} +\Delta _{y - t, T = M,S = m}^{j} } \right)} } \right] \\ \end{aligned}$$

and

$$\begin{aligned} D_{y,T}^{j} & = \phi_{y,T = N} \cdot \left[ {\sum\limits_{t = 1}^{7} {\rho_{t, T = N, s = f} \cdot \left( {O_{y - t, T = N}^{j} +\Delta _{y - t, T = N,s = f}^{j} } \right)} } \right] \\ & \quad + \left( {1 - \phi_{y,T = N} } \right) \cdot \left[ {\sum\limits_{t = 1}^{7} {\rho_{t, T = M, s = f} \cdot \left( {O_{y - t, T = M}^{j} +\Delta _{y - t, T = M,s = f}^{j} } \right)} } \right] \\ \end{aligned}$$

where *φ*_*y*,*T*_ is the proportion of sires and dams of lambs born in year *y* that originate from either the nucleus tier (*T* = *N*) or the multiplier tier (*T* = *M*), and *ρ*_*t*,*T*,*s*_ is the proportion of sires (*s* = *m*) and dams (*s* = *f*) coming from age group *t* in tier *T*. The selection differentials ∆_*y*−*t*,*T*,*s*_ give the superiority of animals of sex *s* in age group *t* that were born in year *y* − *t*, selected to become parents in tier *T* over all animals born that year, and are calculated using selection index theory as described in a later section. For the initial years (*y* − *t* < 0 where 0 is the initial year of recording and selection in the multiplier tier), when dam and sire genetic merit values would be required to be derived from offspring that have not yet been generated, the merit of the missing offspring was calculated by assuming a constant linear rate of genetic progress among age groups. The model does not currently allow optimised selection across age cohorts as it was assumed that the age profile of selected rams is often predetermined due to the need to have a number of mature rams to mate younger ewes, and to provide genetic connectedness among year classes of lambs. In addition, there is an implicit assumption that those rams and ewes mated in any year subsequent to their first mating were kept at random from the ewe and ram lambs originally selected.

Elite rams required for the nucleus and multiplier tiers were sourced from within the nucleus. Ram lambs born and selected in the multiplier tier were used as sires of lambs in the commercial tier. The recorded multiplier tier produced its own ewe replacements where young female candidates were selected based on genetic merit or on their phenotypic performance depending on the scenario under consideration. Replacement nucleus ewes were sourced from within either the nucleus or multiplier tiers, depending on the scenario. The commercial tier produced its own ewe replacements based on traditional non-recording methods.

### Flow of multiplier ram’s genes into the commercial tier

Discounted genetic expressions (DGE) coefficients predict the proportion of genetic superiority that is transmitted to an individual’s descendants through transfer of genes [15]. In the current study, DGE coefficients model the flow of genes from multiplier rams once they enter the commercial flock for mating. We followed the methodology first proposed by McClintock and Cunningham [16], also used by Amer [17] and Berry et al. [18], to predict the timing and frequency of genetic expressions which deliver the ultimate economic benefits at commercial flock level. The expressions of genes in different age groups were calculated using five distinct matrices by adapting the methodology described by Amer [17]. Tables 2 and 3 present the age distribution and survival in the different tiers, assuming a constant age distribution structure of breeding ewes and rams in all tiers. These parameters, used in the calculations of DGE coefficients in the gene-flow model, were obtained from the commercial breeding scheme representing a typical set of farmers within the New Zealand sheep industry.

**Table 2:** Ewe age structure in different tiers of the breeding scheme supporting the commercial tier

Age of ewes (years)	Proportions of ewe age groups in the flock (*ρ*_*s*=*f*_)			Probability of ewe survival to age group *i* given presence in age group 2 (**a**_*i*_)	Probability of a ewe dying or being culled at age *i* (**d**_*i*_)	Prolificacy by age group (Lrp_*i*_)
	Nucl	Mult	Comm	Comm	Comm	Comm
1^a^	0.09	0.00	0.00	1.00	0.00	0.80
2	0.32	0.35	0.30	1.00	0.23	1.49
3	0.25	0.23	0.23	0.77	0.10	1.65
4	0.18	0.18	0.20	0.67	0.17	1.65
5	0.14	0.13	0.15	0.50	0.17	1.65
6	0.01	0.09	0.10	0.33	0.28	1.65
7	0.00	0.02	0.02	0.06	0.05	1.65

Nucl is the nucleus tier of the breeding scheme, Mult is the multiplier tier and Comm is the commercial tier

^a^This refers to the proportion of ewes mated in the first year of age as ewe lambs

**Table 3:** Ram age structure in different tiers of the breeding scheme supporting the commercial tier

Age of rams (years)	Proportions of ram age groups in the flock (*ρ*_*s*=*f*_)			Probability of a ram surviving to age group *i* given presence in age group 1 (*α*_*i*_)		
	Nucl	Mult	Comm	Nucl	Mult	Comm
1	0.35	0.28	0.28	1.00	1.00	1.00
2	0.30	0.27	0.27	0.86	0.95	0.95
3	0.15	0.25	0.25	0.43	0.90	0.90
4	0.10	0.20	0.20	0.29	0.72	0.72
5	0.05	0.00	0.00	0.14	0.00	0.00
6	0.05	0.00	0.00	0.14	0.00	0.00

Nucl is the nucleus tier of the breeding scheme, Mult is the multiplier tier and Comm is the commercial tier

The five expression matrices **D**, **E**, **F**, **G** and **H**, account for the probability (**a**_*i*_) of ewes of all ages surviving to the next age, across the different age groups in time, quantifying the flow of genes from parents to the other age groups. The matrix **D** has dimension *h* × *h*, where *h* is defined in years equivalent to the number of age groups, i.e. *h* = 7. Matrix **D** maps the probability (**a**_*i*_) of a ewe surviving to the next age group in successive years and is defined as:

$${\mathbf{D}}_{i,j} = \left\{ {\begin{array}{*{20}l} {{\mathbf{a}}_{i - j} ,} \hfill & \quad {{\text{for}} \ldots j < i - 1 \ldots {\text{and}} \ldots i - j \le c} \hfill \\ 0 \hfill & { \ldots {\text{otherwise}}} \hfill \\ \end{array} } \right.,$$

where *i* and *j* = 1, … *h*, and *c* is a cull for age threshold. The vectors of increments of genetic superiority, by year *k* of expression for each generation (${\mathbf{g}}_{k}$) for the seven age groups across all cohorts, account for the ewe’s genetic contribution to the progeny and is calculated as:

$${\mathbf{g}}_{k} = \frac{1}{2} \cdot f \cdot {\mathbf{D}} \cdot {\mathbf{g}}_{k - 1} ,$$

where *f* is the number of ewe lambs required as replacements per ewe lambing per year. Aggregate yearly genetic expressions accumulated over the generations are calculated as the sum of vectors, i.e. ${\mathbf{g}}_{sum} = \sum\nolimits_{k = 1}^{7} {{\mathbf{g}}_{k} }$.

Additional expression matrices **E**, **F**, **G** and **H** (all with *h* × *h* dimension) are used to map the occurrence of genes at the birth of a new generation that expresses specific categories of traits over the years of the lives of different classes of animals in each generation. The (*i*, *j*) elements of matrix **E** represent the number of lambs produced for slaughter per ewe within each age group repeated as columns within matrix **E**, but with elements shifted down by one row for successive columns, and are calculated as:

$${\mathbf{E}}_{i,j} = \left\{ {\begin{array}{*{20}l} {{\mathbf{a}}_{i - j} \cdot \left( {{\mathbf{v}}_{i} - f} \right),} \hfill & \quad {{\text{for}} \ldots j < i - 1 \ldots {\text{and}} \ldots i - j \le c} \hfill \\ 0 \hfill & { \ldots {\text{otherwise}}} \hfill \\ \end{array} } \right.,$$

where **v**_*i*_ is the number of lambs weaned at age *i* in years, *c* is a cull for age threshold.The elements of the lambing expressions matrix **F** represent the number of lambs born within each age group, such that:

$${\mathbf{F}}_{i,j} = \left\{ {\begin{array}{*{20}l} {{\mathbf{a}}_{i - j} \cdot {\mathbf{v}}_{i - j} ,} \hfill & \quad {{\text{for}} \ldots j < i - 1 \ldots {\text{and}} \ldots i - j \le c} \hfill \\ 0 \hfill & { \ldots {\text{otherwise}}} \hfill \\ \end{array} } \right.,$$

while **G** contains elements representing proportions of ewes dying or being culled in the different ages (**d**_*i*_) as:

$${\mathbf{G}}_{i,j} = \left\{ {\begin{array}{*{20}l} {{\mathbf{d}}_{i - j} ,} \hfill & \quad {{\text{for}} \ldots j < i - 1 \ldots {\text{and}} \ldots i - j \le c + 1} \hfill \\ 0 \hfill & { \ldots {\text{otherwise}}} \hfill \\ \end{array} } \right.$$

and **H**_*i*,*j*_ describes the expressions of replacement ewes (18 months old), with elements of 1 for *i* = 2 and *j* = *i* + 1, or 0 otherwise.

For breeding rams that are transferred from the multiplier tier to the commercial tier, genetic expressions transmitted via replacement daughters were obtained by multiplying the cumulative genetic superiority that is expressed in each age group (**g**_*sum*_) by the relevant expression matrix of each trait group. These traits were grouped in four vectors denoted **w** which count the number of expressions of the genes of a ewe replacement that enters the flock by itself, and her descendants. The rows of the vector **w** correspond to the year following the birth of the replacement female. Vectors **w** are superscripted for traits that are expressed annually by breeding ewes (**w** ^*A*^), in hogget replacement ewes (**w** ^*H*^), at birth by lambs (**w** ^*L*^), and at slaughter by lambs (**w** ^*S*^), and were calculated as:

$$\begin{aligned}&{\mathbf{w}}^{A} = {\mathbf{D}} \cdot {\mathbf{g}}_{sum} ,\quad {\mathbf{w}}^{H} = {\mathbf{H}} \cdot {\mathbf{g}}_{sum} ,\\ & {\mathbf{w}}^{L} = {\mathbf{F}} \cdot \frac{1}{2 \cdot ls}{\mathbf{g}}_{sum} + k_{L} \quad {\text{and}}\quad {\mathbf{w}}^{S} = {\mathbf{E}} \cdot \frac{1}{2 \cdot ls}{\mathbf{g}}_{sum} + k_{S} \end{aligned}$$

where *ls* is lamb survival from birth to slaughter. Because surplus lambs are generated in the process of breeding replacement ewes, and these lambs express traits at both birth and slaughter, constant adjustment factors *k*_*L*_ (for lambing traits) and *k*_*S*_ (for slaughter traits), are incorporated into the equations for **w** ^*L*^ and **w** ^*S*^, respectively. These adjustment factors give the direct genetic expressions of a slaughter trait per replacement ewe kept, which is calculated by using an adaptation of formulae described in Amer [17], based on the proportion of ewe lambs surviving to slaughter age that are retained as ewe flock replacements (*u*) as:

$$k_{L} = \left( {\frac{1}{ls}} \right)\left( {\frac{1}{2}u} \right)^{ - 1} \quad {\text{and}}\quad k_{L} = \left( {1 - \frac{1}{2}u} \right)\left( {\frac{1}{2}u} \right)^{ - 1} .$$

It was necessary to link expressions by ewe replacements in the various **w** vectors to the number of sires mated in the commercial flock to generate replacements over multiple mating years. To do this, matrices **Z** ^*j*^ for each trait type *j* (lambing, slaughter, annual ewe and replacement ewe traits) with columns made up of the lagged expression vectors for the corresponding trait type were applied. These matrices represent ram matings over successive years assuming they survive, but lagged down one row, for each successive potential year of re-mating. Thus, rows of matrices **Z** correspond to the year *e* following the first mating, and columns *t* correspond to the successive years of mating of the ram. Then, survival of the ram can be taken into account when computing a final vector of commercial trait expressions, and it was convenient to scale these expression vectors so that the sum of the elements in the expression vector equals 1. This procedure was applied to the different trait groups (*j*) and the calculation was:

$$\varepsilon_{e}^{j} = \frac{{\sum\nolimits_{t} {{\mathbf{Z}}_{e,t}^{j} \cdot {\varvec{\upalpha}}_{t} } }}{{\sum\nolimits_{t} {\sum\nolimits_{e} {{\mathbf{Z}}_{e,t}^{j} \cdot {\varvec{\upalpha}}_{t} } } }},$$

where **α**_*t*_ is the probability of a breeding ram surviving in the commercial flock to year *t* after its first year of mating.

Thus, $\varepsilon_{e}^{j}$ is the sum of discounted expressions of trait *j* by a ram from the multiplier tier in each year (*e*) of expression after its first mating in the commercial flock but expressed as a proportion of its total lifetime sum of expressions. The numerator adds up the expressions by year of expression (after first mating), and the denominator standardises the expressions into a proportion by year of expression relative to the overall expressions.

### Selection differentials

This study applied selection index principles to quantify responses to selection based on a pre-determined multiple-trait breeding objective. The definition of the aggregate breeding value of selection candidates, across tiers, was calculated as the sum of the products of economic weights (*ew*_*j*_) of the traits *j* composing the breeding objective (made up of *n* traits), and their respective breeding values (*ebv*_*j*_), and computed by $H = \sum\nolimits_{1}^{n} {\left( {ew_{j} \cdot ebv_{j} } \right)}$, as described by Hazel et al. [19].

Selection differentials were calculated for each of the component traits of the breeding objective described in Table 4. The selection differentials were computed based on deterministic selection index equations [14]. The selection index model predicted index weights, the index additive genetic variance, and the consequent regression coefficients of each component trait on the index. The selection differentials were obtained as the product of the index standard deviation (*σ*_*T*,*s*_), the respective regression coefficients of traits on the index ($b_{T,s}^{j}$) and the selection intensities (*i*) corresponding to a year of the breeding program (*y*), in the tier from which the parents were selected (*T*) and the sex of the parents (*s*), as follows:

$$\Delta _{y,T,s}^{j} = \sigma_{T,s} \cdot b_{T,s}^{j} \cdot i_{y,T,s} .$$

**Table 4:** Heritability (*h* ^2^), genetic standard deviation (*σ*_*g*_), accuracies (*r*), accuracy of genomic prediction (*r*_*GBV*_), economic values (*EV*) and weights (*ew*) for various traits used in the simulation

DGE Trait group^a^	Trait (abbreviation)	Unit	*h* ^2^	*σ*_*g*_	*r*	*r*_*GBV*_	*EV* ($/unit)	*ew* ($/unit)
Slaughter	Carcase weight (CWT)	kg	0.30	1.10	0.60	0.50	2.60	3.74
	Weaning weight (WWT)	kg	0.20	1.57	0.58	0.48	0.93	1.36
Annual	Number of lambs born (NLB)	Lambs	0.10	0.18	0.25	0.54	22.31	22.31
	Ewe mature weight (EWT)	kg	0.45	0.99	0.30	0.50	−0.94	−1.49
	Ewe body condition score (BCS)	Score	0.18	0.30	0.30	–	12.93	12.93
	Survival maternal (SURm)	Lambs	0.05	0.09	0.16	–	52.20	83.78
	Weaning weight maternal (WWTm)	kg	0.10	1.11	0.25	0.47	1.02	1.21
Hogget	Stayability (Stay)	Binary	0.15	0.15	0.41	–	19.28	19.28
Lambing	Lamb survival (SUR)	Lambs	0.01	0.04	0.13	–	52.20	92.46

^a^Traits grouped by animal class that expresses the trait

The regression coefficients are calculated as $b_{T,s}^{j} = \frac{{Cov\left( {I_{T,s} , tbv_{j} } \right)}}{{Var\left( {I_{T,s} } \right)}}$, where *I*_*T*,*s*_ is the index derived by using standard selection index theory and assuming a set of information sources appropriate for selection candidates in tier *T* and of sex *s*, and *tbv* is the true breeding value of trait *j*. The parameters required for the calculation of the regression coefficients and selection differentials are in Tables 4, 5 and 6. Table 4 presents trait economic values (*EV*_*j*_) calculated as the marginal profit per unit change in the trait *j* per animal in the class where the trait is expressed [20]. Economic values were used to calculate benefits of genetic changes in animals that express the relevant trait, whereas economic weights (*ew*_*j*_) were used to define optimal index weights underlying the selection index model, and therefore *EV*_*j*_ incorporated standard DGE coefficients [17] that are used in the national breeding index for dual purpose sheep in New Zealand. Trait accuracies (*r*) and genomic prediction accuracies (*r*_*GBV*_) were also obtained from the New Zealand national genetic evaluation system, Sheep Improvement Limited (SIL). Trait accuracies represent the correlation between estimated breeding values (EBV) and true breeding values. Genomic prediction accuracies represent the correlation between pedigree-based EBV and the genomic breeding values (GBV) estimated based on genomic and phenotypic information from the national reference population [6, 7, 21]. According to Auvray et al. [21], the training set is made up of a mixture of pure and crossbred animals, with Illumina OvineSNP50K BeadChip [50K single nucleotide polymorphism (SNP) chip] genotypes from 13,420 individuals to investigate BLUP with different genomic relationship matrices and SNPs and to predict the GBV of younger animals.

**Table 5:** Genetic (below diagonal) and phenotypic (above diagonal) correlations between traits used in the selection index model

Trait (abbreviation)	WWT	WWTm	NLB	SUR	SURm	EWT	BCS	Stay	CWT
Weaning weight (WWT)	1.00	0.00	0.00	0.00	0.00	0.55	0.00	0.00	0.75
Weaning weight maternal (WWTm)	0.00	1.00	0.00	0.00	0.00	0.00	0.00	0.00	0.00
Number of lambs born (NLB)	0.00	0.00	1.00	0.00	0.00	0.00	0.00	0.00	0.00
Lamb survival (SUR)	0.00	0.00	0.00	1.00	0.00	0.00	0.00	0.00	0.00
Survival maternal (SURm)	0.00	0.00	0.00	0.00	1.00	0.00	0.00	0.00	0.00
Ewe mature weight (EWT)	0.55	0.00	0.00	0.00	0.00	1.00	0.50	0.20	0.75
Ewe body condition score (BCS)	0.00	0.00	0.00	0.00	0.00	0.50	1.00	0.20	0.00
Stayability (Stay)	0.00	0.00	0.00	0.00	0.00	0.20	0.20	1.00	0.00
Carcass weight (CWT)	0.75	0.00	0.00	0.00	0.00	0.75	0.00	0.00	1.00

**Table 6:** Selection proportions and resulting selection intensities for ewe and ram lambs in different tiers

Parameter	Nucleus		Multiplier		Commercial	
	Ewes	Rams	Ewes	Rams^b^	Ewes	Rams^c^
Selection proportion^a^	0.70	0.05	0.80	0.20	0.00	0.30
Selection intensity (*i*)	0.49	2.06	0.35	1.40	0.00	1.16

^a^Proportion of candidates selected to potentially become a replacement ewe or a breeding ram

^b^Rams selected in the nucleus to mate ewes in the multiplier tier

^c^Rams selected in the multiplier to mate ewes in the commercial tier

The methodology modelled genomic selection by defining GBV as additional traits, which are genetically and phenotypically correlated with the traits included in the selection index model (Table 5), similar to methodology described by Dekkers [22]. Because, in practice, GBV for specific traits are expected to be genetically correlated with other traits, the correlations between GBV traits and each other trait was calculated as, $r_{{GBV_{i} ,BV_{j} }} = r_{{BV_{i,} BV_{j} }} \cdot r_{{GBV_{i,} BV_{i} }}$, where $r_{{BV_{i,} BV_{j} }}$ is the genetic correlation between traits *i* and *j*, and $r_{{GBV_{i,} BV_{i} }}$ is the accuracy of genomic prediction for trait *i*, presented in Table 4. The calculations assumed that GBV had a phenotypic standard deviation of 1.0, a heritability of 0.95 and an economic value of 0.

Table 6 presents selection proportions and selection intensities for each sex and tier. These result from replacement policy decisions and the age structure of the flock, which influence the proportions of ewe and ram lamb candidates selected in the different tiers.

### Scenarios

The nucleus animals were assumed to be fully recorded, with phenotypes and parentage or genomic selection assigned to all individuals. In the nucleus tier, live weight at different ages, ultra-sound scanning, body condition score and maternal traits were routinely recorded. Parentage assignment was carried out through DNA testing in all lambs born. Alternatively, when genomic selection was assumed, selection candidates were tested on a 5K SNP chip to determine genomic relationships and SNP profiles. Sires used in the nucleus were assumed to be tested on the 50K SNP chip.

The base scenario assumed no performance recording or genotyping in the multiplier tier. A range of scenarios were compared, which included different combinations of policies for phenotypic recording, DNA parentage, genomic selection, genotyping strategy, and nucleus replacement policy.

Two levels of phenotypic recording policies were evaluated. The “simple” performance recording policy was assigned for recording pregnancy scanning [as a proxy for number of lambs born (NLB)], live weight and body condition score on ewes of all ages, and only weights for lambs, without maternal information. In this case, selection could only take place based on individual performance information, since with no parentage assignment it is impossible to use information from relatives when evaluating candidates in the multiplier tier. The “complete” performance recording policy had the assumption that weaning, slaughter and carcass weights, body condition score, pregnancy scanning results, lambing and weaning rates were recorded on ewes of all ages and their lambs.

In the DNA parentage policy, it was assumed that lambs born in the multiplier tier were either assigned to their dams and sires by DNA testing (Yes), or that there was no DNA parentage assignment (No). On a commercial scale, the advantage of DNA parentage testing is that it is a more practical and accurate method of parentage determination than matching lambs to ewes at birth, which is labour intensive and requires single sire mating groups.

In the genomic selection policy, it was assumed that progeny born in the multiplier flock were genotyped on the 5K SNP chip (Yes), or GS was not applied (No). GS is different to pedigree allocation via DNA, in that it generates higher accuracy of selection than can be achieved through identification of an animal’s relatives via pedigree information. GS is based on knowledge of the relationships between individuals, and between their genotypes (SNP) and phenotypes, established using reference populations.

The genotyping strategy policy also had two alternate levels, “All” and “Selective”, when all lambs born in the multiplier tier were genotyped and when a subset of individuals were genotyped for GS, respectively. In the case where a “Selected” policy is implemented, only physically sound replacement ewes and ram candidates born as twins or triplets could be potentially selected, which resulted in effective genotyping of 47% of the ewe lambs and 28% of the ram lambs born in the multiplier. There are two situations where it may be necessary to genotype all progeny. First, in situations in which phenotypes of the ungenotyped animals may be important to avoid bias in genetic evaluation or to improve selection accuracy, or second, if the turn-around time for genotyping is too slow to allow genotype results to be back in the time period between culling of unsuitable candidates and the final selection of breeding animals.

Alternative replacement policies compared a “Closed” to an “Open” nucleus. In a closed nucleus, candidate ewe lambs were selected only from within the nucleus tier. The alternative policy selected part of the female nucleus replacements, based on truncation selection, from among the top ewes within the multiplier tier (open nucleus).

The different scenarios established the basis for the selection index model which, along with trait specific genetic parameters, was used to estimate the genetic progress attributed to each set of selection strategies. A summary of the simulation scenarios modelled in this study is in Table 7.

**Table 7:** Description of simulation scenarios modelled to the multiplier tier of a multi-tiered breeding scheme

Scenario	Policies				
	Performance recording	DNA parentage	Genomic selection (GS)	Genotyping strategy	Nucleus replacement policy
Base scenario^a^	–	No	No	–	Closed
Pheno + GS	Complete	No	Yes	All	Closed
Pheno + selective GS	Complete	No	Yes	Selected	Closed
Pheno + parentage	Complete	Yes	No	All	Closed
Phenotypic selection	Simple	No	No	–	Closed
Pheno + GS + open	Complete	No	Yes	All	Open
Pheno + parentage + open	Complete	Yes	No	All	Open
GS Only	–	No	Yes	All	Closed
Selective GS Only	–	No	Yes	Selected	Closed

^a^Refers to the base scenario in which no performance recording or genetic merit selection is undertaken in the multiplier flock

The GS Only and the Selective GS Only scenarios assumed that GS genotypes were the unique source of information for selection in multiplier candidates, i.e. not combined with phenotypes. This assumes that a relevant and effective training population for genomic selection is available outside of the multiplier itself, and thus, no investment in recording is required to maintain this training population.

### Genetic lag

The genetic lag between tiers was calculated as the difference in average merit of progeny ($O_{y,T}^{j}$) in the nucleus and the lower tiers at a given point in time. Average genetic lag at year 20 in a higher (*T* = *H*) and lower (*T* = *L*) tier for trait *j* were calculated as:

$$Lag_{{T = H, T^{\prime } = L}}^{j} = \frac{{O_{y = 20, T = H}^{j} - O_{{y = 20, T^{\prime } = L}}^{j} }}{{b_{{T^{\prime } = L}}^{j} }},$$

where $b_{{T^{\prime } = L}}^{j}$ is the annual rate of genetic progress of trait *j*, in the lower tier, between years 20 and 30, a period when the rate of genetic progress had stabilized.

### Genetic trend in the commercial flock

The model predicted specific genetic trends for different traits, after the implementation of the alternative scenarios. The breeding program was simulated over 40 years from the moment when performance recording and genotyping were implemented in the multiplier tier. The reference trend for comparison was based on the selection practices from the base scenario.

The annual sum of expressions ($EBV_{y}^{j}$) for trait *j* across the different age groups, i.e. seven ewe age groups, of the commercial flock in a given year *y*, was computed as:

$$EBV_{y}^{j} = \sum\limits_{e = 1}^{7} {\left[ {\left( {O_{T = M, y - e}^{j} +\Delta _{T = M, s = m, y - e}^{j} } \right) \cdot \varepsilon_{e}^{j} } \right]} .$$

where $O_{T = M, y - e}^{j}$ is the average genetic merit of offspring born in the multiplier tier in year *y* from the start of a new recording strategy, after year *e* following the first mating of a ram in that tier of the breeding structure, ∆ is the selection differential of the trait *j* in the respective year in the given *T* tier, for the selected males *s* = *m*, and $\varepsilon_{e}^{j}$ is the discounted expression of trait *j* in age group *e* in the commercial flock, expressed as a proportion of their total lifetime sum of expressions, as described in the equation that calculates $\varepsilon_{e}^{j}$ as the sum of DGE expressed as a proportion of its total lifetime sum of expressions.

### Economic evaluation

The economic impact of implementing recording efforts was calculated as the product of the direct trait expressions ($EBV_{y}^{j}$), their economic values (*ev*_*j*_), and the number of animals affected (*n* ^*j*^, described in Table 8) within the breeding scheme. Additional revenue was summed across tiers for the different scenarios. The additional revenue (*R*_*y*_) realised in the commercial flock in year *y* was calculated as:

$$R_{y, T = C} = \sum {EBV_{y}^{j} \cdot ev_{j} \cdot n^{j} } .$$

**Table 8:** Prices of recording associated components and number of animals tested in the different scenarios

Cost component	$/unit	Animals tested (*n*)^a^							
		Scenarios^b^							
		Pheno + GS	Pheno + selective GS	Pheno + par	Phenotypic selection	Pheno + GS + open	Pheno + par + open	GS only	Selective GS only
DNA Parentage Test	20.00	0	0	10,507	0	0	10,507	0	0
5K SNP Test	50.00	10,507	3921	0	0	10,507	0	10,507	3921
EID	1.50	10,507	10,507	10,507	10,507	10,507	10,507	10,507	10,507
Recording	2.00	17,507	17,507	17,507	17,507	17,507	17,507	0	0
Genetic evaluation	2.00	17,507	17,507	17,507	0	17,507	17,507	17,507	17,507

^a^Number of animals tested, identified, recorded and evaluated annually in different scenarios

^b^Scenario are described in Table 7

In the multiplier tier, the additional revenue realised from trait improvements after implementation of recording efforts was calculated as:

$$R_{y, T = C} = \sum {\left( {D_{y, T = M}^{j} \cdot ev_{j} \cdot nd_{T = M} } \right) \cdot \rho_{d}^{j} } + \sum {\left( {EBV_{y, T = M}^{j} \cdot ev_{j} \cdot no_{T = M} } \right) \cdot \rho^{j} } ,$$

where $D_{y}^{j}$ and $EBV_{y}^{j}$ are the average genetic merit of dams and offspring respectively, born in tier *T* of the breeding structure for trait *j* in year *y*, while *nd*_*T*=*M*_ and *no*_*T*=*M*_ are the respective numbers of dams and offspring in the multiplier tier, $\rho_{d}^{j}$ and *ρ* ^*j*^ are the proportion of dams and offspring expressing the *j*th trait, after discounting the percentage of ewes mated to terminal sires and lambs sold as store (Table 1).

The net profit relative to the base scenario was calculated from the extra revenue minus the cost as:

$$\pi_{y} = \sum\limits_{y} {\left( {R_{y, T = M} + R_{y, T = C} - C_{y}^{TOT} } \right) \cdot \left( {\frac{1}{1 + r}} \right)^{y} } ,$$

where *r* is a 7% annual discount rate and *C* ^*TOT*^ is the total recording and selection related costs in year *y* calculated as:

$$C_{y}^{TOT} = C_{y}^{dna} + C_{y}^{eid} + C_{y}^{rec} + C_{y}^{ge} ,$$

where $C_{y}^{dna}$ are the parentage and genomic selection costs, $C_{y}^{eid}$ is the cost of electronic identification, $C_{y}^{rec}$ are the estimated recording or direct measurement costs and $C_{y}^{ge}$ is the genetic evaluation cost in scenarios for which parentage or genotypes were available.

These cost components were calculated as:

$$\begin{aligned} & C_{y}^{dna} = \left( {\$_{dna,y} \cdot n_{dna,y} } \right) + \left( {\$_{SNP,y} \cdot n_{SNP,y} } \right),\quad C_{y}^{eid} = \left( {\$_{eid,y} \cdot n_{eid,y} } \right), \\ & \quad C_{y}^{rec} = \left( {\$_{rec,y} \cdot n_{rec,y} } \right);\quad {\text{and}}\quad C_{y}^{ge} = \left( {\$_{ge,y} \cdot n_{ge,y} } \right), \end{aligned}$$

where $ represents the price of the different component costs, i.e. DNA parentage and SNP tests ($_*dna*_), electronic identification ($_*eid*_), phenotype recording practices ($_*rec*_) and genetic evaluation ($_*ge*_), which were assumed to remain constant over time, and *n* is the number of animals tested in year *y*. Table 8 presents these parameters for the different scenarios.

The discounted cumulative net present value of additional profit (*CNPV*) was calculated as the difference between the profit after implementation of recording efforts (*π*′), from year 0 to year *y*, for any given multiplier recording scenario expressed as a deviation from the profit obtained in the base scenario (*π*), computed as:

$$CNPV_{y} = \sum\limits_{i = 1}^{y} {\pi_{i}^{\prime } - \pi_{i} } .$$

## Results

### Economic impact

Relative to the base scenario, the annual additional revenue that was generated by performance recording in the multiplier, grew steadily from 2 years after the introduction of new scenarios, and stabilized to a constant value after 11 years in all scenarios. The Pheno + GS + open scenario presented the largest benefits. This scenario reached constant annual revenues of $724 K from year 11 onwards. The next largest benefits were obtained in scenarios Pheno + GS, and Pheno + Selective GS. Both scenarios reached constant annual revenues of $713 K, also from year 11 onwards. The next best results arose from selection on phenotypes and parentage, represented in scenarios Pheno + parentage + open and Pheno + parentage, which produced annual increases in revenue of $576 K and $553 K, respectively. The annual revenues of genomic selection only scenarios, GS only and Selective GS only, stabilized at $530 K. Phenotypic selection alone had the smallest benefits, with stabilized marginal revenue of $13 K, also achieved after 11 years.

The cost of recording efforts in the multiplier tier of the breeding scheme had the biggest impact during establishment in year 1 because of the implementation costs when all breeding ewes were genotyped, or simply identified for phenotypic selection. In the following years, costs stabilized due to a fixed number of animals being tested, recorded and evaluated, and due to the assumption that relative prices remained constant throughout the simulation planning horizon.

The phenotypic selection strategy did not include parentage assignment or genomic selection, and therefore, had the smallest overall annual cost among all scenarios, at $108 K after completion of the establishment phase. Nevertheless, the very modest benefits that arose from this strategy did not offset the low costs. The annual cost of the Pheno + parentage scenario was $318 K. The annual cost of the Selective GS only was $290 K. The cost of the Pheno + selective GS was $329 K per year, while the annual cost for GS only was $594 K, and Pheno + GS was $634 K. Because fewer animals were tested in selective GS genotyping scenarios, these had lower costs when compared to the equivalent scenarios in which all lambs born were genotyped. There was no difference in costs between open and closed nucleus versions in Pheno + GS and Pheno + parentage scenarios, which were otherwise identical strategies.

In scenarios which involved both performance recording and parentage, the cost of parentage assignment through DNA was the largest cost component, comprising 70% of the total recording cost. Genomic testing was the most significant cost component in GS scenarios (from 64 to 88%), when compared to the cost of trait measurements (10 to 36%), electronic identification (9 to 27%) and genetic evaluation (10 to 15%).

### Scenario comparison and cost-benefit analysis

**Fig. 1:**
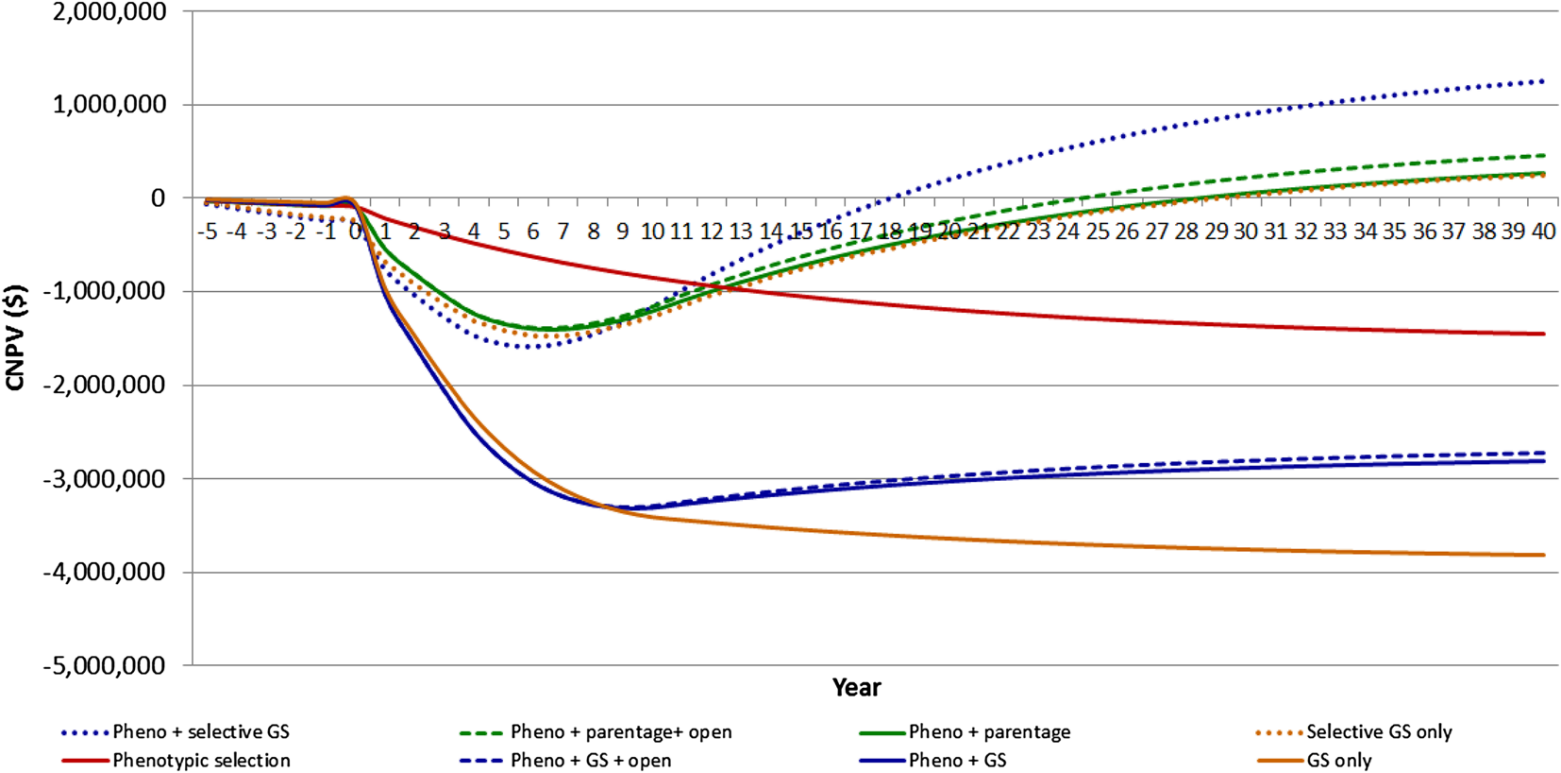
Cumulative net present value (*CNPV*) of simulation scenarios, relative to the base scenario, in different selection strategies of the multiplier tier of the breeding scheme. Scenarios based on different selection strategies, described as: *Pheno* + *GS* phenotypic recording and genomic selection, *Pheno* + *parentage* phenotypic recording and parentage for pedigree selection, *Pheno* + *GS* + *open* phenotypic recording, genomic selection and the open nucleus, *Pheno* + *parentage* + *open* phenotypic recording, parentage for pedigree selection and the open nucleus, *Pheno* + *selective GS* phenotypic recording and alternative genotyping for genomic selection of physically sound candidates with potential to become replacement ewes and future only, *Selective GS only* genomic selection without performance recording in the multiplier by genotyping for genomic selection only physically sound candidates with potential to become replacement ewes and future rams. *Phenotypic selection* selection based on phenotypes only, *GS only* GS without performance recording in the multiplier

Figure 1 presents the cumulative net present value (*CNPV*) that results from implementation of recording procedures in the multiplier tier over successive years, relative to the base scenario. Scenario Pheno + selective GS achieved breakeven the earliest, after 18 years. The profitability of this scenario in year 30 was about $903 K. Pedigree selection represented by the Pheno + parentage scenarios, in both open and closed nucleus, reached breakeven in years 25 and 29, respectively, producing *CNPV* of $226 K and $58 K in year 30. Scenarios with complete phenotyping and GS genotyping of all lambs did not achieve breakeven within the simulated horizon. The *CNPV* reaches breakeven only in scenarios in which phenotypes were combined with parentage information or when only a selected subset of candidates was genotyped.

**Fig. 2:**
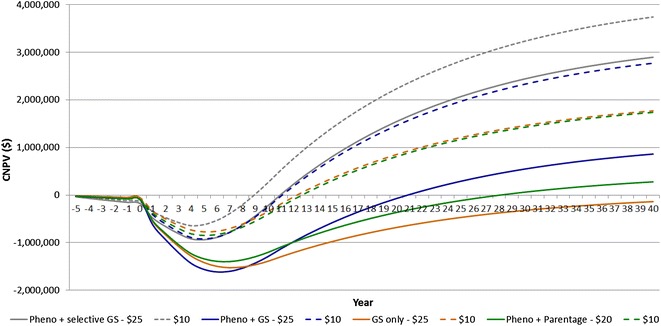
Cumulative net present value (CNPV) of alternative selection strategies, assuming recording the multiplier tier under genotyping test prices at $10 and $25. See description of scenarios in Fig. 1 and details in Table 7

In general, the cost of parentage testing and genomic selection greatly influenced the *CNPV* of recording the multiplier tier. Under the current costs of genotyping, the most profitable selection strategies will take 18 to 29 years to achieve financial breakeven, thus resulting in long periods of large financial deficits. However, if genotyping costs decrease to $25 or less, then recording a sheep multiplier tier in the conditions included in the present study becomes more attractive. For instance, under lower GS genotyping costs of $25, as opposed to $50 per test, the long-term profit of the Pheno + GS scenario increased to $549 K in year 30, compared to a *CNPV* of −$2800 K. The breakeven point and *CNPV* of a range of scenarios under lower parentage test prices are in Fig. 2.

### Selection differentials

Changes in selection differentials that result from the selection index model, which assumed performance recording implemented in year 1, were the main drivers of variation in benefits among scenarios. All selection differentials for the “base scenarios” are 0 for animals within the multiplier tier as there was no information to select on. The set of differentials for rams selected in the recorded multiplier tier to mate commercial breeding ewes are in Table 9. For clarity, trait selection differentials in the nucleus for ewes and rams contribute to overall genetic progress, and differentials in the multiplier and commercial tiers contribute to decreased genetic lag between higher and lower tiers.

**Table 9:** Selection differentials of recorded multiplier breeding rams for profit traits (units/year) in the different scenarios

Trait (abbreviation)	Unit	Pheno + GS^a^	Pheno + parentage	Phenotypic selection	Pheno + GS + open	Pheno + parentage + open	GS only^b^
Carcass weight (CWT)	kg	0.674	0.598	0.293	0.665	0.581	0.645
Weaning weight (WWT)	kg	0.868	0.759	0.334	0.857	0.739	0.835
Number of lambs born (NLB)	Lambs	0.053	0.020	0.016	0.054	0.023	0.056
Ewe mature weight (EWT)	kg	0.548	0.698	0.297	0.540	0.678	0.342
Ewe body condition score (BCS)	Score	0.058	0.042	0.000	0.057	0.041	0.032
Survival maternal (SURm)	Lambs	0.008	0.010	0.000	0.010	0.012	0.000
Weaning weight maternal (WWTm)	kg	0.090	0.040	0.000	0.093	0.047	0.087
Stayability (Stay)	Binary	0.012	0.018	0.000	0.010	0.018	0.001
Lamb survival (SUR)	Lambs	0.003	0.004	0.000	0.003	0.004	0.000

Scenario description in Fig. 1 and details in Table 7

^a^Selection differentials used in Pheno + GS and Pheno + selective GS scenarios

^b^Selection differentials used in GS only and Selective GS only scenarios

Differentials for number of lambs born and weaning weight maternal were larger, and differentials for ewe mature weight were smaller, for GS scenarios when compared to selection based on phenotypes only, or on phenotypes and parentage. The phenotypic selection scenario produced the smallest selection differentials, which reflects the low index accuracy in commercial rams selected on phenotypes only, in the multiplier tier (Table 10). Selection differentials of ewes selected in the recorded multiplier differed between the open and the closed nucleus. The differences were limited to BCS, weaning weight maternal and Stay, which were bigger in the open nucleus scenarios when compared to closed nucleus scenarios. These moderate differences in replacement policy caused modest changes in differentials of rams selected in the multiplier tier to be mated in commercial flocks.

**Table 10:** Index accuracies of selection for breeding ewes and rams mated in different tiers in alternative scenarios

Scenario	Nucleus		Multiplier		Multiplier to commercial
	Ewes	Rams	Ewes	Rams	Rams
Pheno + GS	0.43	0.49	0.43	0.49	0.49
Pheno + selective GS	0.43	0.49	0.43	0.49	0.49
Pheno + parentage	0.36	0.38	0.32	0.38	0.37
Phenotypic selection	0.36	0.38	0.18	0.21	0.14
Pheno + GS + open	0.53	0.49	0.53	0.49	0.49
Pheno + parentage + open	0.46	0.38	0.45	0.38	0.38
GS only	0.44	0.49	0.32	0.33	0.33
Selective GS only	0.44	0.49	0.32	0.33	0.33

Scenario description in Fig. 1 and details in Table 7

Table 10 presents the index accuracies of selection in the different tiers and scenarios. The accuracies were based on the number of phenotypic records of the different information sources, and underpin the genetic progress achieved through the range of selection scenarios.

### Genetic contributions

**Fig. 3:**
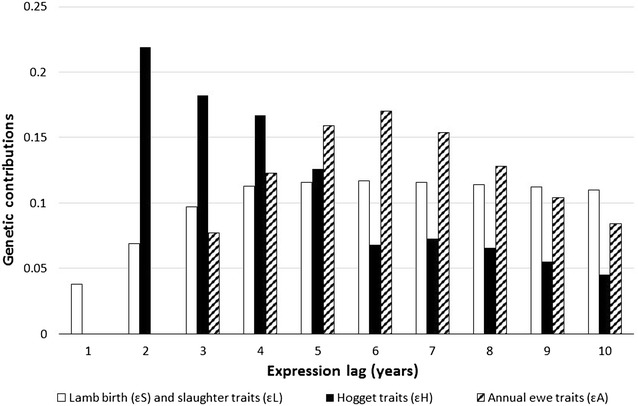
Proportions of genetic contributions for different trait categories from ram selection in the multiplier flock as expressed in the self-replacing commercial ewe flock in years after recording and genotyping were introduced. Number of years from when a ram is first mated in the commercial tier. *ɛ* ^*L*^ = lamb birth traits, *ɛ* ^*S*^ = lamb slaughter traits, *ɛ* ^*H*^ = hogget traits and *ɛ* ^*A*^ = annual ewe traits

The set of genetic expressions (ε), used in the calculation of gene flow from the multiplier tier through to the commercial tier, are in Fig. 3. The results show that traits of the lamb at birth and at slaughter were expressed earlier, followed by ewe hogget traits and traits expressed annually by adult ewes, respectively. After the use of a ram, there will still be an impact of that selection expressed 10 years later through ewes that stay in the flock, and their female descendants. In addition, while slaughtered lamb traits and ewe annual trait expressions decrease slowly after peaking at 6 years, hogget trait expressions peak in the second year and drop fast after 5 years. It could be expected that the “open multiplier” presents a different timing and extent of expressions, given the difference in the age distribution of ewes. However, the replacement of nucleus ewes with older proven multiplier replacements did not affect the genetic expressions at the commercial level.

Ram and ewe selection on performance and genetic records in the multiplier tier resulted in a lift in genetic merit in both multiplier and commercial tiers. This superiority was expressed as the rate of genetic progress in units of the different breeding objective traits, and as the average genetic lag between the different tiers, expressed in years, for the different traits.

### Genetic lags

Table 11 shows the average genetic lag between the nucleus and the commercial tier for the various traits in year 20, in the different scenarios. Trait lags in the commercial tier are represented for the base scenario (*Base*) and after recording was implemented (*With recording*) in the multiplier tier. A reduction from 1 to 4 years in the lag between the nucleus and the commercial tier was achieved with implementation of recording efforts in the multiplier tier. Phenotypic selection caused a reduction of less than a year, while DNA parentage and GS reduced the lag between the nucleus and the commercial tier in more than 3 years. Phenotypic selection alone did not reduce the genetic lag for BCS, SURm, WWTm and Stay. Consequently, phenotypic selection of these traits in the multiplier tier had modest economic impact when compared to the base scenario. The small reduction in genetic lag observed for lamb survival, especially in Phenotypic selection, GS only and Selective GS only scenarios, reflects its low rate of genetic progress and/or the small difference in merit between tiers.

**Table 11:** Genetic lag (years) between nucleus and the commercial tier of the breeding scheme by year 20 in different scenarios

Trait	Base scenario	Pheno + GS	Pheno + selective GS	Pheno + parentage	Phenotypic selection	Pheno + GS + open	Pheno + parentage + open	GS only	Selective GS only
	Base	With recording							
CWT	10.6	7.6	7.6	7.8	9.4	6.7	6.7	7.9	7.9
WWT	10.6	7.7	7.7	7.8	9.5	6.7	6.7	8.0	8.0
NLB	11.0	8.2	8.2	8.3	8.8	7.4	8.0	8.0	8.0
EWT	10.8	7.8	7.8	8.0	9.8	7.1	7.1	9.8	9.8
BCS	10.6	7.2	7.2	6.8	10.6	7.2	7.2	9.5	9.5
SURm	11.5	8.5	8.5	8.2	11.5	8.5	8.0	14.0	14.0
WWTm	11.5	8.3	8.3	8.3	11.5	7.5	8.2	8.4	8.4
Stay	9.2	4.8	4.8	4.8	9.2	5.2	4.8	11.0	11.0
SUR	10.9	7.0	7.0	7.1	10.6	6.1	6.0	13.0	13.0

See scenarios description of Fig. 1 and details in Table 7

The reduction in genetic lag for the different traits varied considerably across scenarios. This was the result of differences in rates of genetic progress between traits in different scenarios. The difference in estimated trait merit reflects the 8 to 40% higher rates of gain in commercial progeny with the recorded multiplier scenarios, compared to the base scenario. By year 20, scenarios assuming GS in the multiplier tier presented the largest difference, i.e. more progress, in genetic merit of traits such as CWT, WWT, and SURm. The DNA parentage strategy resulted in more progress of traits such as NLB, BCS, WWTm and Stay in the commercial tier, when compared to the base scenario.

## Discussion

The objective of this study was to assess the potential benefits that arise from introducing performance recording, parentage testing and genomic selection to the multiplier tier of a multi-tiered breeding scheme, which supplies improved rams to a large number of commercial ewes. The 2 to 7% gains in rate of genetic progress resulting from these recording efforts reflect the importance of trait expressions in the much larger number of animals at the base population of the breeding scheme.

The model considered the cost-benefit of information in commercially-managed sheep flocks. It demonstrated the effectiveness of performance records when combined with parentage information (gains of 5 to 7%), and when associated with genomic selection (gain of 6%), from the perspective of genetic progress. Genomic selection can have a dramatic effect on the reliability of breeding values, especially for sex-limited traits with high accuracy in some livestock industries [23]. Sise and Amer [24] predicted that the sheep meat industry in New Zealand could achieve a 5% increase in the annual rate of genetic progress by adopting multi-trait selection indexes with genomic selection in the nucleus tier. In this study, we also predicted the largest annual benefits, i.e. additional revenue, in scenarios assuming DNA parentage and those assuming GS.

These results were likely due to the greater amount of information used in GS scenarios, which combine genome-based and pedigree-based relationships, as described by Dekkers [22] and Tusell et al. [25]. The results of this study were similar to those from Horton et al. [8] which estimated higher long-term net dollar gains from the use of genomic selection in a range of scenarios assuming various levels of prediction accuracy. The authors described how different tiers were able to benefit from the extra genetic gain derived from genomic testing, and how the degree of multiplication is important when calculating benefits which should offset the increased costs of genomic testing. Horton et al. [8] considered that recording the multiplier tier was not cost-effective because of the prohibitive testing prices. In this study, benefits for commercial flocks were conditional on assumed accuracies of prediction, which ultimately depend on the amount of data measured in the reference population, especially when animals with genotypes and records of their own were available.

According to Pickering et al. [9], the rate of genetic gain can be lifted when selecting young rams on a dual purpose index (a New Zealand sheep national selection index [20]) with genomic information, from which the majority of the benefit comes from the increased accuracy of breeding value for sex-limited traits and those recorded later in life. However, the study of Pickering et al. [9] was based on direct benefits from recording only in the breeder flocks, and did not consider benefits from recording the multiplier tier.

This study found large differences in benefits and costs across scenarios, and in the timing at which cumulative benefits offset the cumulative costs. Under current genotyping prices, the implementation of the GS policy with higher revenue was not cost-effective and both the time to breakeven and the long-term *CNPV* were not economically attractive. High implementation costs will likely moderate the practical application of the selection in GS scenarios that were investigated in this simulation study, particularly the cost of parentage testing and genomic selection. The cost of genomic technology is dropping rapidly and the expectation is that prices will further decrease, with encouraging possibilities developed in the area of genotyping by sequencing (GBS) [26]. Reduction in genotyping costs and policies which restrict genotyping to animals for which the most benefit can be generated, will likely be a key to achieve higher adoption of recording efforts and genotyping among producers. According to van der Werf et al. [27], genotyping approximately 20% of a young sire crop, would result in close to maximum overall additional benefits of genomic selection. In this study, reduced genotyping costs from either lower prices and/or reduced proportion of genotyped selection candidates changed the *CNPV* and repositioned scenarios accordingly. We demonstrated this when cost-effectiveness was achieved through the selective genotyping policy (Fig. 1), and through the sensitivity analysis, in which genotyping prices were reduced by 50% or more, with increased profitability (Fig. 2). The long-term nature of the benefits that are associated with informed selection of multiplier candidates demonstrates the critical importance of achieving lower costs of information. This is particularly important for large-scale commercial operations that need to adopt genomic technology on a wider scale, than nucleus operations.

The structure of the breeding scheme that was simulated in this study assumed a larger than necessary multiplier tier, relative to the size of nucleus and commercial tiers. This resulted in economic losses when genotyping all lambs, compared to pedigree selection, even with the larger selection differentials (Table 9). In a recorded multiplier scenario, which initially incurs higher recording and genotyping costs, these losses could be avoided by having a smaller multiplier flock while still allowing sufficient selection intensity so that genetic progress can be maintained. In a different breeding structure, with a smaller relative size of the multiplier population, it is likely that genomic selection policies, with a larger proportion of genotyped animals, would produce positive results.

The selection scenarios that resulted in the earliest and largest financial returns were parentage assignment assuming current DNA testing prices. Under lower genotyping costs, the scenarios that combined performance recording with genomic selection ranked as the most profitable. Information on genotypes or parentage becomes critical to achieving significant rates of genetic progress, because the low heritability of maternal traits (i.e. traits measured late in life) limits the effectiveness of phenotypic selection. Phenotypic information is only useful for genetic evaluation if records can be combined with parentage and/or genomic relationship information; otherwise it is very difficult to estimate genetic merit with sufficient accuracy to be useful.

The selection policy simulated in the GS only scenario was not profitable under the accuracy of genomic predictions assumed in this study. In practice, it is difficult to achieve a high GS accuracy for traits with low heritability, such as maternal traits, which depend on more phenotypic data to derive prediction equations of high accuracy [6]. Higher prediction accuracies can be achieved through GS if an appropriate reference population exists to predict the phenotype of individuals from their genotype. Information collected in the multiplier tier could be useful to increase the reference population. This is because multi-tiered breeding schemes normally have large numbers of related animals with potential to generate performance data and genomic information in different tiers. The accuracy of genomic prediction can be increased if the reference population includes information with the highest average genetic relationship to the reference set [28]. Further investigation into the additional contribution of genomic selection to the accuracy of prediction in the nucleus might provide additional support for recording and genotyping in the multiplier tier.

In addition to overall profitability and time to breakeven, higher chances of adoption of profitable selection strategies might depend on the existence of infrastructure to facilitate performance recording. Infrastructure to collect and store records requires investment in tracking individual animal production and development of uniform procedures for measurement, sampling, testing, data analysis and selection of candidates. Identifying and keeping the most productive commercial ewes for further lambing opportunities decreases the number of replacement ewes required and could potentially increase the flock overall lifetime performance [29]. In this study, the open nucleus resulted in only modest changes in *CNPV* when compared to the closed nucleus. There was not enough additional genetic gain to offset recording costs, or to compensate for eventual increases in generation interval. According to Garrick et al. [12], if the additional information from performance recording results in delays and increased generation interval, this would erode benefits from higher accuracy that are created by the introduction of older multiplier ewes as nucleus flock replacements.

An open nucleus policy might be more beneficial in the presence of genotype by environment interaction (G×E) where the nucleus tier is not fully representative of the commercial environment, and or if inbreeding management is an issue in the breeding program. In the presence of G×E, goal traits expressed in the commercial tier may differ from the reference trait expressed in the nucleus tier. Phenotypes and genotypes recorded in the multiplier flock, which is frequently maintained in a commercial environment, could be used to predict genetic merit in multi-tier breeding populations. This could inform the extent to which genomic accuracy of prediction in the nucleus tier could impact trait expressions in the commercial tier, or vice versa.

In summary, recording in the multiplier tier reduced the lag between the nucleus and the commercial tier by 2 to 3 years. This benefit has an important economic impact for commercial lamb production. Similar results were suggested by Hill [15] in pig populations using multiplier tiers, and by Horton et al. [8] in sheep. According to Bichard [11], genetic lags can be considerable and their actual size is determined by the annual rate of progress in the nucleus tier, flock age structure in the lower tiers, and origin and degree of selection intensity. Bichard [11] reported a lag of 11 years between nucleus and commercial tiers in three-tiered sheep breeding schemes. According to Blair and Garrick [13], the typical genetic lag in the New Zealand sheep industry is between 5 and 8 years, based on a two-tier breeding structure, and assuming that rams transferred from the nucleus to the commercial tier are the average of those born in the same year in the nucleus.

Breakeven and overall profitability depended highly on key pieces of information that enabled higher accuracy of prediction and consequently the highest level of genetic progress. Because net benefits were relatively modest when compared to the scale of investment required, adoption of recording efforts and genotyping by producers is likely to be low at the base levels of DNA test cost modelled here. Reductions in DNA technology costs, or recording policies that either mitigate problems with G×E interactions or facilitate accurate selection in nucleus candidates via DNA parentage (eliminating pedigree errors) and genomic prediction, may change the likelihood of the adoption of recording in multiplier tiers in the future. Nevertheless, scientific modelling of breeding schemes can assist in quantifying the genetic and economic impacts of selection alternatives. An additional selection scenario, which would be worthwhile to investigate and has not been included in the current study, is the presence of G×E and its impact on the prediction accuracy of genomic selection.

## Conclusions

Our findings demonstrate that performance recording and parentage assignment in the multiplier tier can generate long-term economic benefits in multi-tiered breeding programs. Implementing recording in the multiplier tier reduces the long genetic lag between the nucleus and commercial tiers. Such recording is justified if the breeding scheme captures the benefits through more profit generated in the commercial tier. The investment in phenotyping is only worthwhile if parentage or genomic information is also available. Genomic selection also has the potential to significantly increase the benefits of recording, especially under reduced genotyping costs or when a subset of candidates is tested, as opposed to all lambs born. Finally, genomic selection policies in multiplier tiers might be feasible in the near future, given the expected reduction in genotyping costs, which are critical drivers of the magnitude of benefits, and of the time required to break-even investments in recording.
